# Remote Mobile Outpatient Monitoring in Transplant (Reboot) 2.0: Protocol for a Randomized Controlled Trial

**DOI:** 10.2196/26816

**Published:** 2021-10-22

**Authors:** Kevin R Murray, Farid Foroutan, Jennifer M Amadio, Juan Duero Posada, Stella Kozuszko, Joseph Duhamel, Katherine Tsang, Michael E Farkouh, Michael McDonald, Filio Billia, Edward Barber, Steven G Hershman, Mamatha Bhat, Kathryn J Tinckam, Heather J Ross, Christopher McIntosh, Yasbanoo Moayedi

**Affiliations:** 1 Temerty Faculty of Medicine University of Toronto Toronto, ON Canada; 2 Ted Rogers Centre of Excellence in Heart Function Peter Munk Cardiac Centre University Health Network Toronto, ON Canada; 3 Ted Rogers Computational Program University Health Network Toronto, ON Canada; 4 Ajmera Transplant Centre University Health Network Toronto, ON Canada; 5 Pattern Health Durham, NC United States; 6 Division of Cardiovascular Medicine Department of Medicine Stanford University Stanford, CA United States; 7 Division of Gastroenterology & Hepatology Department of Medicine University Health Network Toronto, ON Canada; 8 Division of Nephrology Department of Medicine University Health Network Toronto, ON Canada

**Keywords:** mobile health, telemonitoring, transplantation, wearable sensors, solid organ transplant

## Abstract

**Background:**

The number of solid organ transplants in Canada has increased 33% over the past decade. Hospital readmissions are common within the first year after transplant and are linked to increased morbidity and mortality. Nearly half of these admissions to the hospital appear to be preventable. Mobile health (mHealth) technologies hold promise to reduce admission to the hospital and improve patient outcomes, as they allow real-time monitoring and timely clinical intervention.

**Objective:**

This study aims to determine whether an innovative mHealth intervention can reduce hospital readmission and unscheduled visits to the emergency department or transplant clinic. Our second objective is to assess the use of clinical and continuous ambulatory physiologic data to develop machine learning algorithms to predict the risk of infection, organ rejection, and early mortality in adult heart, kidney, and liver transplant recipients.

**Methods:**

Remote Mobile Outpatient Monitoring in Transplant (Reboot) 2.0 is a two-phased single-center study to be conducted at the University Health Network in Toronto, Canada. Phase one will consist of a 1-year concealed randomized controlled trial of 400 adult heart, kidney, and liver transplant recipients. Participants will be randomized to receive either personalized communication using an mHealth app in addition to standard of care phone communication (intervention group) or standard of care communication only (control group). In phase two, the prior collected data set will be used to develop machine learning algorithms to identify early markers of rejection, infection, and graft dysfunction posttransplantation. The primary outcome will be a composite of any unscheduled hospital admission, visits to the emergency department or transplant clinic, following discharge from the index admission. Secondary outcomes will include patient-reported outcomes using validated self-administered questionnaires, 1-year graft survival rate, 1-year patient survival rate, and the number of standard of care phone voice messages.

**Results:**

At the time of this paper’s completion, no results are available.

**Conclusions:**

Building from previous work, this project will aim to leverage an innovative mHealth app to improve outcomes and reduce hospital readmission in adult solid organ transplant recipients. Additionally, the development of machine learning algorithms to better predict adverse health outcomes will allow for personalized medicine to tailor clinician-patient interactions and mitigate the health care burden of a growing patient population.

**Trial Registration:**

ClinicalTrials.gov NCT04721288; https://www.clinicaltrials.gov/ct2/show/NCT04721288

**International Registered Report Identifier (IRRID):**

PRR1-10.2196/26816

## Introduction

Solid organ transplant (SOT) is the standard therapy for select patients with severe end organ dysfunction. During the transition from pre- to posttransplant, recipients are introduced to a number of new medications and medical issues. In light of this complexity, early hospital readmission is common, ranging from 18% to 47% [1-3]. In addition to an increased burden on the health care system, early hospital readmission is associated with increased patient morbidity and mortality [4]. Although some readmissions are unpredictable and unavoidable, as many as half of early readmissions may be preventable [4].

Mobile health (mHealth) technologies such as smartphones and wearable devices hold promise to reduce hospitalization and improve patient outcomes through real-time monitoring, prompting timely clinician intervention that cannot be replicated in traditional outpatient care [5]. A recent systematic review showed mHealth apps improve patient self-management and reduce rates of rehospitalization in adults with cardiovascular disease [6]. Previous work from our group has shown that mHealth technology is safe, feasible, and associated with a 50% relative risk reduction in rehospitalization in adults with heart failure [7]. We anticipate that SOT recipients would also benefit from improved monitoring and removal of communication barriers as the most common reasons for readmission (eg, infection, rejection, elevated blood pressure, and metabolic derangements such as renal, glucose, and electrolyte abnormalities) and mortality may be mitigated by clinical intervention [1,2,5,8]. Additionally, medication adherence is critical in transplant patients to prevent graft rejection [9,10]. Enhanced and clear communication is likely to lead to improved patient outcomes.

Over the past decade, the number of SOTs in Canada has increased by 33% [11]. As transplant program volumes continue to grow, the need to reduce hospital readmissions is critical both for patients and to avoid straining the health care system. Building upon prior research in which the safety and feasibility of mHealth technologies in adult patients with heart transplants was demonstrated [12], larger scale clinical data is needed to determine whether this technology reduces hospital readmissions.

In the transplant population, it is likely that remote monitoring will improve medication adherence/adjustments and will allow for identification of early decompensation and reduce preventable hospital readmissions. Thus, this study will determine if an innovative mHealth intervention designed to improve patient-clinician communication reduces unnecessary hospital readmission and visits to the emergency department (ED) and transplant clinic when used in addition to the standard of care telephone communication system. We will also incorporate clinical and continuous ambulatory physiologic data collected as part of the mHealth intervention to develop machine learning (ML) algorithms capable of identifying early indicators of adverse outcomes in adult patients with heart, kidney, and liver transplants.

Therefore, we hypothesize that the delivery of personalized communication using an mHealth app will improve patient self-management, resulting in a reasonable reduction in preventable hospital readmission and unscheduled visits to the ED and transplant clinic of 50%. With tailored communication through the mHealth app, we expect fewer standard of care phone messages for patients in the intervention group, and patients with higher activity levels (average daily step count) pretransplantation will have lower index hospitalization length of stay. Finally, the large data set collected from this study will allow novel ML-derived risk prediction models to more accurately predict adverse outcomes (eg, organ rejection, infection, and death) compared to conventional regression models.

## Methods

### Overview

**Figure 1:**
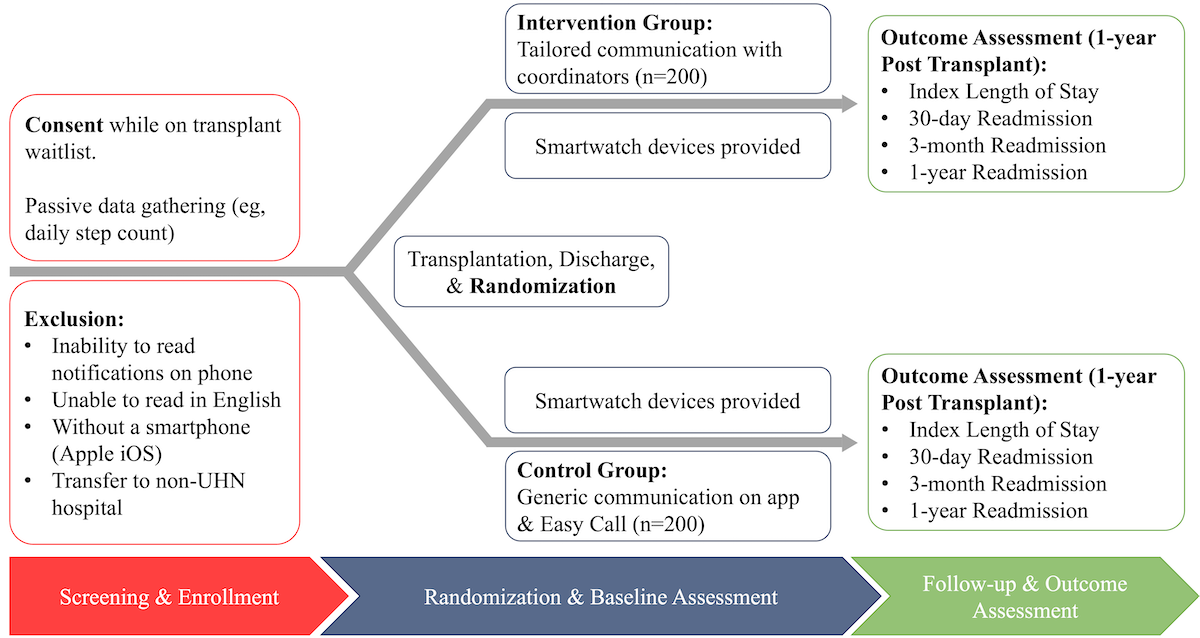
Proposed study flowchart. UHN: University Health Network.

Remote Mobile Outpatient Monitoring in Transplant (Reboot) 2.0 is a registered (ClinicalTrials.gov NCT04721288) two-phased single-center study. It has also been approved by the University Health Network (UHN) Research Ethics Board (REB 20-6082.0.1). The first phase will consist of a 1-year concealed randomized controlled trial of 400 adult SOT (heart, kidney, or liver) recipients. Currently, the Ajmera Transplant Centre at the UHN (a large academic teaching site in Toronto, Canada) uses paper medical administration records to alter immunosuppressive medications, and patients generally record their vitals by pen and paper, both of which may be error prone. Additionally, health care providers and patients with transplants rely on a telephone-based (“Easy Call”) method of communication, wherein patients leave a message for their health provider when issues arise. The integration of medication and vitals tracking, as well as patient-provider communication into a single mHealth app may decrease errors and improve patient outcomes. To test this, participants will be randomized to receive either personalized communication using an mHealth app in addition to the Easy Call system (intervention group) or Easy Call standard of care (control group; see Figure 1). In the second phase of Reboot 2.0, the data set from the 400 patients with SOTs collected as part of phase one will be evaluated in an attempt to identify early markers of rejection, infection, and graft dysfunction posttransplant.

### Participants

The UHN in Toronto, Canada conducted over 700 transplants in 2019, including approximately 225 liver transplants, 225 kidney transplants, and 40 heart transplants. Based on this distribution, we expect to recruit 150 to 200 liver transplant, 150 to 200 kidney transplant, and 40 to 50 heart transplant recipients from the SOT waiting list at the UHN. Inclusion criteria include heart, kidney, or liver transplant recipients; age ≥21 years; the ability to use a smartphone (Apple iOS); and English speaking. Exclusion criteria include patients with poor health literacy (defined as a reading level less than grade 5), transfer to a non-UHN hospital for follow-up and management, and an inability to follow instructions from the mHealth app.

### Screening and Recruitment

Patients on the UHN transplant waiting list will be screened by study personnel for participation in this study. Consent and enrollment will be completed while participants are still on the transplant waiting list. Prior to discharge from the index hospitalization, patients will be randomized to either the intervention or control group. All patients will be given an Apple Watch at the time of discharge from their index hospitalization to record continuous ambulatory physiologic data. The use of smartwatch technology will allow autonomous step count tracking and rapid clinician intervention in response to patient inactivity, heart rate monitoring, heart rate variability, intermittent electrocardiography, and the development of robust ambulatory physiologic data that can power predictive ML algorithms for adverse patient outcomes.

### Randomization

Concealment of allocation will be ensured by the use of a centralized 24-hour internet-based computerized randomization system. Participant will be the unit of randomization, and to protect against imbalance in known prognostic factors between groups, stratified randomization will be used. Stratification will be based on age (ie, 18-31 years, 31-50 years, or >50 years of age), organ transplanted (ie, heart, kidney, or liver), listing status (ie, urgent transplant or nonurgent transplant), and history of organ replacement therapy (ie, left ventricular assist device or dialysis). Block randomization, using four blocks, will ensure balance between participants randomized to the intervention and control groups.

### Intervention

Both groups will have access to the Reboot 2.0 mHealth app and receive generic notifications (eg, for achieving a daily step goal) as well as the option to log vital signs (eg, blood pressure, heart rate, weight, and temperature); however, only participants in the intervention group will receive tailored communication. This aspect of the intervention is designed to improve patient care by providing organ-specific educational material, a medication list, and weekly updates on fitness status (eg, heart rate and step count) with the overall goal of improving patient self-care and management. This will also be supported through the delivery of personalized notifications (eg, app use incentives, appointment reminders, medication changes, and clinical care updates) through the app. The use of reminder notifications and the smartphone calendar will help avoid missed tests and appointments in the intervention group. More importantly, the intervention group will have access to active communication with the transplant care team, including the ability to send asynchronous messages for nonurgent issues. Asynchronous messaging will be available as an integrated function within the mHealth app, accessible by the patient on their smartphone device and by the care team through the app’s provider dashboard. The care team will respond to messages within 48 hours (the same time frame as the standard of care system) and copy message conversations from the provider dashboard to the electronic medical record, avoiding the need for verbal message transcription. This will reduce the need for patients and clinicians to leave messages on the standard of care system and therefore improve patient-provider communication. We expect that improving patient education and self-management, and alleviating communication barriers will facilitate earlier recognition of decompensation and avoid preventable hospitalizations and ED visits.

### Standard of Care (Easy Call System)

Easy Call is the standard of care in the Ajmera Transplant Program within the UHN. This system allows transplant health care providers to leave messages for the patients regarding changes in their medication and appointment reminders. Patients, in turn, can also leave messages for health care providers should they require further clarification on their care. The Easy Call system will be available to both arms of this study.

### Outcomes

The primary outcome for this study will be any unscheduled hospital admission or ED and unscheduled transplant clinic visit post discharge from the index admission during which the patients received their organ transplant. Readmissions and unscheduled ED/transplant clinic visits will be captured at 30 days, 3 months, and 1 year after organ transplant. A Central Adjudication Committee (CAC) composed of two transplant physicians from each organ group (ie, heart, kidney, and liver) will review each reported readmission to determine if they are study events and the most responsible diagnosis for readmission. Secondary outcomes will be assessed to capture participant illness experience and further quantify between-group differences in recovery from index transplantation. These will include patient-reported outcomes using validated self-administered questionnaires (see next section), 1-year graft survival rate, 1-year patient survival rate, number of Easy Call interactions, and index length of stay compared to pretransplant activity level (ie, step count). The CAC will also review all reported secondary outcomes and cases of graft loss or patient death to adjudicate the cause. Any disagreements within the CAC will be resolved by consensus.

### Patient-Reported Outcomes

A health outcomes questionnaire (EQ-5D) and the Patient-Reported Outcomes Measurement Information System (PROMIS) Global Health Scale will be used to assess patient-reported outcomes. The EQ-5D is a comprehensive, compact health status classification and health state preference system. This questionnaire is widely used and has demonstrated validity and sensitivity in many populations [13-15]. PROMIS is a set of person-centered measures that evaluates and monitors physical, mental, and social health in adults and children. It can be used with the general population and individuals living with chronic conditions [16,17]. Both the EQ-5D and PROMIS questionnaires will be completed by participants at minimum 30 days, 3 months, and 1 year after SOT.

### Study Follow-up

All participants will be followed at set intervals (approximately 30 days, 3 months, and 1 year from transplant), with participant health status and outcomes recorded at each point. A 1-year follow-up period was chosen as the study period as most patient challenges occur in the first year following transplant [8]. This time frame is the period of maximal benefit to patients and care providers when we anticipate the majority of unplanned but potentially avoidable readmission occurring.

### ML Approach

Clinical and physiologic data captured in phase one will be continuously streamed from the Reboot 2.0 mHealth app on each participant’s smartphone to Pattern Health’s centralized secure server hosted by Amazon Web Services in the United States. This centralized data lake will be used to develop ML algorithms in the second phase of the study to better interpret raw patient data. Phase one data will consist of routine clinical data taken at baseline and regular follow-up appointments including demographics, comorbidities (eg, chronic kidney disease, diabetes, hypertension, obesity, and longitudinal changes in weight over time), metabolic scale body measurements, and laboratory and imaging investigations; ambulatory physiologic data collected via the Apple Watch (eg, vital signs and activity level); and laboratory and test results entered into the mHealth app (eg, blood glucose measurement or insulin administration). Additionally, details regarding the immunosuppression regimen used and longitudinal changes made will be incorporated. Some episodes of rejection, particularly in patients with liver transplants, may be managed as an outpatient, resulting in modifications to the immunosuppression regimen over time. Medications used for treatment of comorbidities (eg, diabetes, hypertension, or viral hepatitis) will also be taken into consideration (see Multimedia Appendix 1). These data will be automatically streamed into the centralized data lake (in the case of Apple Watch and patient-reported data) or extracted from the participant’s medical record.

Categorical measurements (eg, gender) will be one-hot encoded [18] prior to normalization and temporal adjustment. Specifically, all participant demographic variables will be independently normalized to have mean zero and SD of one. Each feature with missing values will have a corresponding binary variable added denoting if the variable was observed or not. EQ-5D data will be coded into the five dimensions of health as five distinct features for analysis and following established guidelines for normalization [19]. The PROMIS Global Health scale will be coded using a similar approach. All other variables will be independently normalized to have a mean of 0 and an SD of 1. Measurement of mean, SD, maximum, and minimums will be recorded over fixed intervals, and the number of most recent intervals precluding an event will be included in the model (eg, the average heart rate per 3-day window taken over 12 days yields 4 distinct measurements). Missing measurements will be counted per interval as an additional feature. For each patient window, this will create a fixed length feature variable that can be directly included and compared across the statistical and ML models. The interval width and number of intervals will be determined experimentally within the training data set. Following data processing, self-normalizing neural network [20], long short-term memory [21], random forest model [22], and extreme gradient boosted decision tree [23] approaches will be applied on a training portion of the data set to assess risk factors for the development of transplant outcomes. Early ML models will support the development of a ranked features list of clinically relevant variables, which will be used to inform subsequent model development.

The accuracy of each model (discrimination and calibration) and their relative performance (net-reclassification index) will be assessed in a portion of the data set reserved for testing. The development of predictive ML algorithms for adverse patient outcomes will allow for future personalized posttransplant medicine and early clinical intervention based on patient-specific data.

### Statistics

Currently at the UHN, approximately 20% of patients are readmitted to the hospital within 30 days post SOT. In general, these patients are readmitted following presentation to either the ambulatory transplant clinic or ED. Although we do not have data on the number of patients that present to the ambulatory transplant clinic within 30 days post SOT, previous work has shown ED visits at 30 days to vary greatly between SOT groups (eg, as low as 17% for patients with kidney transplants, and as high as 44% for patients with liver transplants) [24,25]. Given this variability, we hypothesized a conservative estimate of 20% for our 30-day composite end point. To observe a 50% relative risk reduction, from 20% to 10%, for the 30-day composite end point, at an alpha of .05 and power of 0.8, 199 participants will need to be recruited for each group.

We will evaluate all outcomes using the intention-to-treat analytical approach. A Cox proportional hazard model will be used to evaluate the effect of the mHealth app on posttransplant unplanned readmission and ED and unscheduled transplant clinic visit rates (primary outcome). Results of this analysis will be reported as hazard ratios with 95% CIs. Kaplan-Meier curves will be used to depict freedom from unplanned readmission. Subsequent to running the Cox proportional hazard model, a logistic regression model will be used to analyze unplanned readmission rates at the prespecified time points of 30 days, 3 months, and 1 year. Results of the logistic regression model will be reported using odds ratios and 95% CIs.

Generalized linear models to estimate the mean difference in the intervention compared to the control group will be used to estimate the impact of personalized communication through the mHealth app on posttransplant patient-reported outcomes (secondary outcomes).

To avoid spuriously inflated estimates of treatment effect, an interim analysis will be conducted when 50% of participants are enrolled and have completed follow-up. The results of this analysis will be reviewed by an independent data monitoring committee. The O’Brien-Fleming stopping rule will be used based on our primary outcome and requires a *P*≤.007 [26]. This conservative rule will avoid premature trial termination unless a large treatment effect is observed.

## Results

Participant enrollment is expected to begin mid-2021. Data collection is anticipated to be completed by late 2023. At the time of this paper’s completion, no results are available.

## Discussion

Reboot 2.0 will build on previous pilot work that demonstrated the safety and feasibility of an mHealth intervention in adult patients with heart transplants [12]. It will be the first study to examine the clinical impact of this mHealth app on a large group (n=400) of diverse (heart, kidney, and liver) SOT recipients. In particular, the clinical trial phase of this research project will determine if leveraging an innovative and patient-centric mHealth app and smartwatch technology reduces hospital readmission and improves patient-reported outcomes as well as patient and graft survival in this population. If so, the mHealth app may be mobilized on a larger scale to reduce the burden on the health care system of a growing patient population. The results of this phase may be limited by its single-center design; however, the Ajmera Transplant Centre at the UHN is the largest in North America and therefore will allow for a diverse and representative participant population.

Phase two of Reboot 2.0 is particularly novel in that it will result in the development of a data registry from 1 year of clinical data, patient-reported data collected through the mHealth app, and ambulatory physiologic monitoring using the Apple Watch from 400 patients with SOTs. This will allow us to develop predictive ML algorithms to better predict adverse health outcomes in these patients, and the subsequent application of these algorithms will allow for more personalized transplant medicine and tailored clinician-patient interactions.

## Appendix

Multimedia Appendix 1 Summary of study measurements to be used in machine learning model development.

## Abbreviations

CAC: Central Adjudication Committee
ED: emergency department
mHealth: mobile health
ML: machine learning
PROMIS: Patient-Reported Outcomes Measurement Information System
Reboot: Remote Mobile Outpatient Monitoring in Transplant
SOT: solid organ transplant
UHN: University Health Network
