# Supplementary material for: Remote Mobile Outpatient Monitoring in Transplant (Reboot) 2.0: Protocol for a Randomized Controlled Trial
Source: JMIR Res Protoc. 2021 Oct 22;10(10):e26816. doi: 10.2196/26816 (PMC8571683; doi:10.2196/26816)
Supplement: Multimedia Appendix 1 [file resprot_v10i10e26816_app1.docx]

Supplemental Table 1. Summary of study measurements to be utilized in machine learning model development.

|  | Assessment Timepoint | Assessment | | | | |
| --- | --- | --- | --- | --- | --- | --- |
| Patient Demographics | Baseline | Age | Gender | Race | Native language | English speaking |
|  |  | Postal Code | Income | Living arrangement | Caregiver requirements | Reason for organ transplant |
|  |  | Waitlist status | Type of organ transplant | Medication | Comorbidities | Functional class |
|  |  | Index hospital length of stay | Index complications |  |  |  |
| Anthropometrics | Baseline, 3, 6, 9, 12 months | Height | Weight | Body Mass Index | Waist circumference |  |
| Laboratory Investigations | Standard of Care | Hemoglobin | WBC count | Neutrophil count | Lymphocyte count | Platelet count |
|  |  | Sodium | Potassium | Calcium | Creatinine | eGFR |
|  |  | Anti-rejection medication trough level | Donor specific antibodies | LDL | Total cholesterol | HDL |
|  |  | TG | non-HDL | HbA1C | Fasting glucose | Urine protein |
|  |  |  |  |  |  |  |
| Physiologic Metrics | Daily | Heart rate | Low heart rate event | High heart rate event | Irregular heart rhythm event | Resting heart rate |
|  |  | Walking heart rate | Heart rate variability | Oxygen Saturation | Body temperature | Mean blood pressure |
|  |  | Systolic blood pressure | Diastolic blood pressure | Respiratory rate | VO_2_max | Blood glucose |
|  |  | Electrodermal activity | Step count | Distance walking/running | Distance cycling |  |
|  |  | Active energy burned | Swimming stroke count | Distance swimming | Distance downhill snow sport | Basal energy burned |
|  |  | Flights climbed | Exercise time | Stand hour | Stand time | Number of falls |
| Patient Health Questionnaires | Baseline, 1, 3, 12 months | EQ-5D | PROMIS Global Health Scale |  |  |  |
| Weekly Check-in Questionnaires | Weekly | Anti-rejection missed dose | Moderate-severe dysfunction | Infection requiring hospital/ER | New/worse graft dysfunction | Unscheduled health visit |
|  |  | ER visit | Admission |  |  |  |

WBC – white blood cell; eGFR – estimated glomerular filtration rate; LDL – low-density lipoprotein; HDL – high-density lipoprotein; TG – triglyceride; HbA1C – hemoglobin A1C; VO_2_max – maximum oxygen uptake; EQ-5D - EuroQol-5 Dimension; PROMIS - Patient-Reported Outcomes Measurement Information System.
